# Effects of chemotherapeutic agent bendamustine for non-hodgkin lymphoma on spermatogenesis in mice

**DOI:** 10.7555/JBR.31.20170023

**Published:** 2018-05-18

**Authors:** Min-bo Zang, Qiao Zhou, Yun-fei Zhu, Ming-xi Liu, Zuo-min Zhou

**Affiliations:** State Key Laboratory of Reproductive Medicine, Department of Histology and Embryology, Nanjing Medical University, Nanjing, Jiangsu 211166, China.; State Key Laboratory of Reproductive Medicine, Department of Histology and Embryology, Nanjing Medical University, Nanjing, Jiangsu 211166, China.; State Key Laboratory of Reproductive Medicine, Department of Histology and Embryology, Nanjing Medical University, Nanjing, Jiangsu 211166, China.; State Key Laboratory of Reproductive Medicine, Department of Histology and Embryology, Nanjing Medical University, Nanjing, Jiangsu 211166, China.; State Key Laboratory of Reproductive Medicine, Department of Histology and Embryology, Nanjing Medical University, Nanjing, Jiangsu 211166, China.

**Keywords:** Non-Hodgkin lymphoma, chemotherapy, bendamustine, mice, spermatozoa, toxicology

## Abstract

Non-Hodgkin lymphoma (NHL) is one of the most common cancers affecting men of reproductive age. The high response rate of bendamustine as first-line treatment for NHL, coupled with young age of patients, makes elucidation of the impact of treatment on male reproduction important. Our aim was to determine the effects of bendamustine on male reproduction by animal model. Male mice were treated with bendamustine (40 mg/kg) through tail vein injection while cisplatin was given as a standard (3 mg/kg) through intraperitoneal injection. After 3 weeks, bendamustine induced weight loss and sperm morphology abnormalities were compared to the control. Additionally, sperm with folded tails were the most frequent abnormality in bendamustine-treated mice. But the mechanism of sperm abnormality induced by bendamustine remains to be elucidated. These results indicate bendamustine may affect spermatozoa of patients who have been treated for NHL.

## Introduction

Non-Hodgkin lymphoma (NHL) is a heterogeneous group of lymphoid malignancies accounting for a significant portion of cancers occurring in children, adolescents and young adults^[1]^. The overall incidence of NHL increases steadily with age and is predominantly higher in males. The increase has been dramatic in patients aged 10–29 years, averaging 4%–19% per year over 25 years^[2]^ Predominant NHL in adolescents and young adults include Burkitt's lymphoma (BL), lymphoblastic lymphoma (LL), diffuse large B cell lymphoma (DLBCL), anaplastic large cell lymphoma (ALCL) and primary mediastinal B cell lymphoma (PMBL)^[1]^. Among them, DLBCL is the most common NHL in adolescence and adulthood^[3]^, accounting for approximately 40%^[2^,^4]^ and 30%^[5]^, respectively, of new diagnoses. Recently, with an overall response rate (ORR) of over 86%^[6]^, bendamustine based therapy showed modest activity in patients with relapsed and refractory DLBCL^[7]^. The high response rate makes the post-treatment quality of life of DLBCL patients a concern, and because of patients undergoing chemotherapy for NHL are usually of reproductive age, consideration of the impact of the treatment on fertility and reproductive function has become increasingly important.

Bendamustine is a water-soluble, bifunctional alkylating agent that also has potential antimetabolite properties and only partial cross-resistance with other alkylators^[8]^. This anticancer agent consists of an alkylating nitrogen mustard group bound to a purine-like benzimidazole ring, and because of this unique bifunctional structure the bendamustine activity profile is significantly different from classical alkylators^[6]^. Although the precise mechanism of action has not been elucidated yet^[6]^, it is known that bendamustine activates the DNA-damage stress response, induces apoptosis, inhibits mitotic checkpoints, and induces mitotic catastrophe. Moreover, bendamustine differs from other alkylators in the type of DNA repair pathways activated. Together, these differences may explain the efficacy of bendamustine observed in a variety of clinical settings^[9]^. While the mechanism of action and cytotoxicity of bendamustine have been extensively studied, we still know little about the effects of bendamustine on the male reproductive system. The clinical studies published so far have reported fairly low or mild toxicity of bendamustine-containing regimens^[9]^. In general, the most common toxicities of bendamustine involve hematological events such as anemia, leucopenia, neutropenia or thrombocytopenia or non-hematological toxicities related to bendamustine treatment include nausea, infections, fatigue, constipation, diarrhea, headache and vomiting^[6]^. However, there is a wide concern that exposure to alkylating agents could have adverse effects on spermatogenesis; paternal exposure to an alkylating agent may alter germ cell quality and finally disrupt embryo development^[10^–^11]^.

Here, we hypothesized that the chemotherapeutic regimen bendamustine is deleterious to the production of spermatozoa, to their morphology and motility. To test this hypothesis, we characterized a mouse exposure model designed to evaluate the effects of bendamustine, in doses analogous to those given to humans, on the male reproductive system. Another alkylating agent, cisplatin, which has been reported with severe reproductive toxicity in mice and rats^[12^–^13]^, was used as a positive control.

## Materials and methods

### Chemicals

Bendamustine was purchased from Medchem Express LLC., USA and cisplatin was purchased from Sigma Chemical Co., USA.

### Animals and treatment

Specific pathogen-free (SPF) C57BL/6 male mice (eight weeks of age) were purchased from Shanghai SLAC Laboratory Animal Co., Ltd. The animals were housed at a constant temperature (20°C- 22°C) at 50%–70% humidity with a 12 hours light: 12 h dark photoperiod. Mice were provided with food and water *ad libitum*. All animal studies were conducted in accordance with the principles and procedures outlined in the Guide to the Care and Use of Experimental Animals prepared by the Nanjing Medical University Council on Animal Care.

Males were randomly divided into four groups of five mice each. In the first two groups, male mice were treated with bendamustine (40 mg/kg) or saline through tail vein injection on day one and two for a total of three weeks. Mice treated with bendamustine served as the bendamustine group, while mice treated with saline served as the control group. The dose regimen of bendamustine was chosen based on the standard dose given to humans (120 mg/m^2^)^[14]^, adjusted for surface area according to the following formula: f x mg/kg= mg/m^2^, where f equals 3.0 for mice^[15]^. In the next two groups, male mice were treated with cisplatin (3 mg/kg) or saline through intraperitoneal injection five days a week for a total of three weeks; mice treated with cisplatin served as the cisplatin group.

### Tissues and sperm collection

At the end of 3 weeks of treatment, male mice were sacrificed by cervical dislocation. The testes were removed, weighed, and fixed in Modified Davidson's Fluid (MDF). The epididymides were removed, trimmed free of fat, and sectioned into the initial segment, caput, corpus, and cauda regions. Unilateral cauda epididymidis was finely minced in fresh phosphate-buffered saline (PBS). The minced tissue was left for 5 minutes on ice with agitation to allow the spermatozoa to disperse and then filtered through a nylon strainer. Spermatozoa were subsequently washed three to five times by centrifugation with a hypotonic buffer (0.45% NaCl) to lyse any contaminated cells and finally washed in PBS. Spermatozoa were then frozen with liquid nitrogen until further use.

### Testicular histopathology

Modified Davidson's Fluid (MDF) fixed testes were then excised, post fixed for an additional 24 hours in the same fixative, dehydrated, and embedded in paraffin for histological examination. Paraffin embedded testis tissues were cut into 5 μm sections. Testis cross sections were then stained with periodic acid-Schiff-hematoxylin (PAS-H) staining kit (Nanjing Senbeijia Biological Technology Co., China). Images were captured with a Zeiss Axio Imager A1 microscope and processed with Adobe Photoshop.

### Indirect immunofluorescence

Paraffin embedded testis tissues were cut into 5 μm sections. Sections were mounted on slides, deparaffinized, and rehydrated. After blocking in 1% bovine serum albumin (BSA) for 2 hours, sections were then incubated overnight at 4°C with polyclonal goat IgG human PLZF antibody (AF2944, R&D Systems, USA) at 1:500 dilution in the blocking solution. The following day, sections were then incubated with an anti-mouse IgG secondary anti-goat Alexa 555 (A-21432, Life Technologies, USA) at a 1:1,000 dilution for 2 hours at room temperature. Images were captured with a Zeiss LSM 700 laser scanning confocal microscope and processed with ZEN lite 2012 (blue edition) software.

### Spermatozoal concentration, motility and morphology

Mature sperm from mice of each group were obtained by making small incisions throughout the cauda epididymis, followed by extrusion and suspension in culture medium (human tubal fluid (HTF) media, Irvine Scientific, USA). Sperm samples (10 μL) were used for computer-assisted semen analysis (CASA) detection (Hamilton-Thorne Research, Inc., USA). Spermatozoal concentration and motility parameters for the experimental and control groups were then measured and analyzed.

Spermatozoa from the cauda epididymis were then spread on slides, and fixed with 4% paraformaldehyde followed by H&E staining as described above for morphological observation.

### TUNEL assay

The TUNEL assay for apoptotic cell detection was performed using the TUNEL BrightRed Apoptosis detection kit (A111-01, Vazyme Biotechnology, China) according to the manufacturer's instructions. Images were captured with a Zeiss LSM 700 laser scanning confocal microscope and processed with ZEN lite 2012 (blue edition) software. The apoptotic indices were then determined by calculating the ratio of the total number of TUNEL-positive cells/number of counted seminiferous tubules.

### Serum testosterone, FSH and LH assessment

Serum concentrations of follicle-stimulating hormone (FSH), luteinizing hormone (LH) and testosterone were measured by enzyme-linked immune sorbent assay (ELISA) as described in the instructions provided by the manufacturer (Nanjing Jiancheng Bioengineering Institute, China).

### Statistical analysis

The differences between the treatment and control groups were analyzed using one-way ANOVA. Values of *P* 0.05were considered to be statistically significant. All data represent the mean±standard error of the mean (SEM).

## Results

### Effects of bendamustine on bodyweight

**Fig.1:**
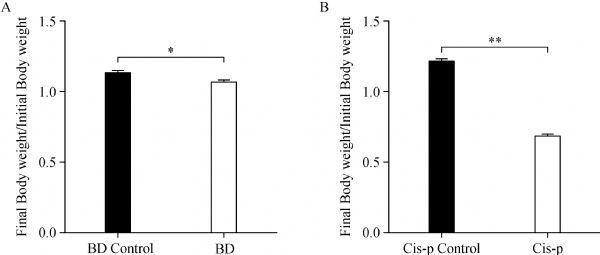
Body weight changes over the course of the three week treatment.

After three weeks of treatment, there was a 5.7%decrease of body weight gain in the bendamustine treated mice compared to the control mice (***Fig. 1A***). One mouse from the cisplatin treated group died during treatment, the remaining mice showed a dramatic43% decrease in body weight compared to thecontrol mice (***Fig. 1B***).

### Effects of bendamustine on testis weights and histology

**Fig.2:**
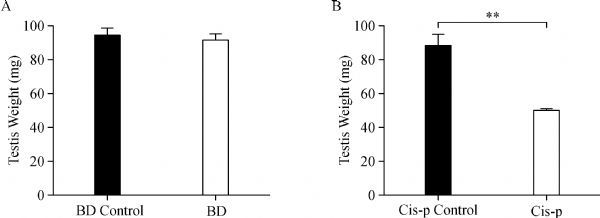
Weights of testis after three weeks of treatment.

The testis of each male mouse was weighed at the end of treatment, and showed changes consistent with changes in body weight. Significant differences were not found in testis weights between the bendamustine treated mice and control mice (***Fig. 2A***). A dramatic 36%decrease was observed in testis weights of cisplatin treated mice compared to the control mice (***Fig. 2B***).

**Fig.3:**
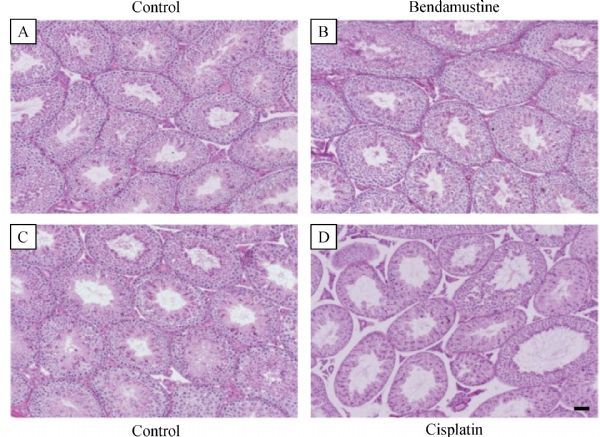
Histopathological examination of testis seminiferous epithelium with PAS-hematoxylin staining.

**Fig.4:**
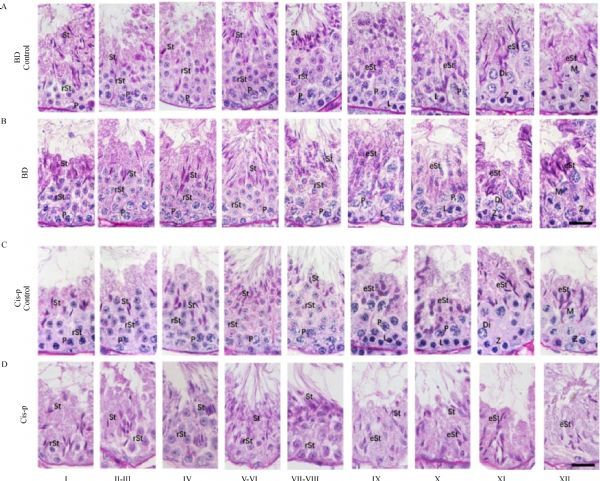
PAS-hematoxylin-stained mouse testis showing different stages of spermatogenesis.

The testis cross-sections of each male mouse were evaluated after three weeks of treatment. There was no significant difference in the cross-sections of the testis between the bendamustine treated group and the control group (***Fig. 3A*** and ***B***). While the testis cross-sections of cisplatin treated mice were characterized by severe atrophy and early germ cell–depleted seminiferous epithelium compared to controls, consistent with decrease in testis weights (***Fig. 3C*** and ***D***). From stage I to stage XII, the depletion of early germ cells was also observed in the seminiferous epithelium of cisplatin treated mice (***Fig. 4D***). Moreover, in stage VIII and IX, unreleased mature spermatids were also observed in the lumen of the seminiferous epithelium in cisplatin treated mice (***Fig. 4D***), reflecting a decrease in the concentration of spermatozoa obtained from the distal cauda epididymidis of mice in the cisplatin treatment group.

### Effects of bendamustine on quantification of spermatogonial stem cells

**Fig.5:**
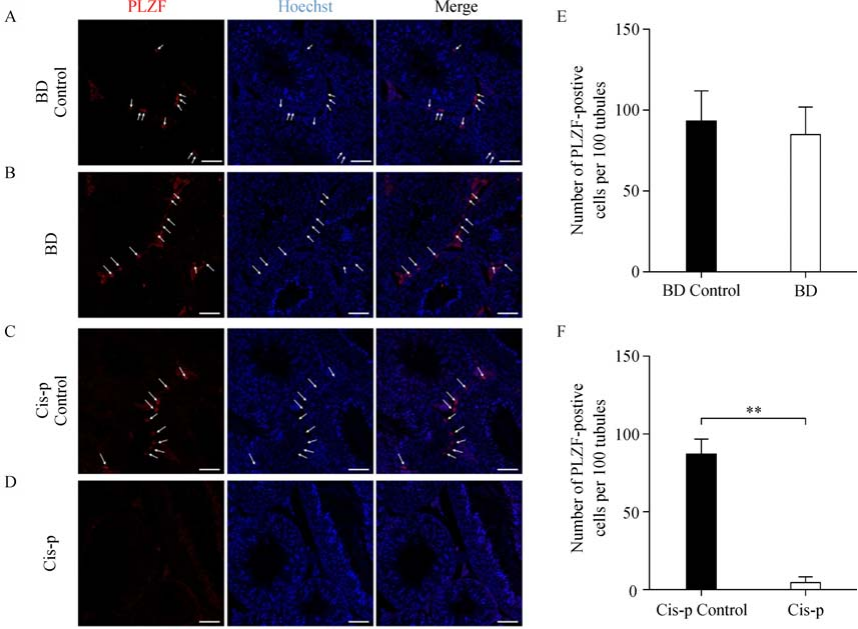
Immunofluorescence localization of PLZF expression in the testis of male mice.

*Via* immunostaining of PLZF, we investigated the impact of these anticancer agents on the population of undifferentiated spermatogonia. We found that cisplatin treatment drastically reduced the number of spermatogonial stem cells (SSCs) in the seminiferous epithelia of the testis, approximately by 93.6% when compared to the control group (***Fig. 5D***, ***E*** and ***F***). However, there was no significant difference in the number of SSCs between the seminiferous epithelia of the testis of bendamustine treated mice and control mice (***Fig. 5A***, ***B***, and ***C***).

### Effects of bendamustine on spermatozoal concentration, motility, and morphology

**Fig.6:**
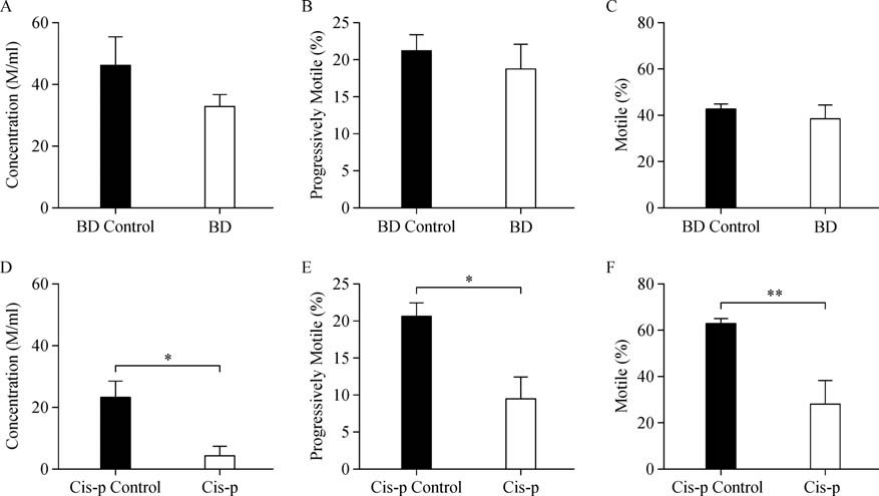
Effects of alkylating agents on the concentration of spermatozoa, percentage of progressively motile spermatozoa, as well as the percentage of motile spermatozoa obtained from the distal cauda epididymidis of mice.

After the course of treatment, we examined spermatozoal concentration and motility via the computer-assisted sperm analysis system (CASA). There were no significant changes in sperm concentration, percentage of sperm motility, progressive motility between bendamustine treated mice and control mice (***Fig. 6A***, ***B***, and ***C***). While in the groups of cisplatin treated mice and control mice, the concentration of spermatozoa in the drug treated mice was reduced by more than 80% when compared to the control mice (***Fig. 6D***). CASA of sperm from the epididymal cauda showed that the mean percentage of motile spermatozoa and progressively motile spermatozoa was more than 30% and 10% lower, respectively, when compared to the controls (***Fig. 6E*** and ***F***).

**Fig.7:**
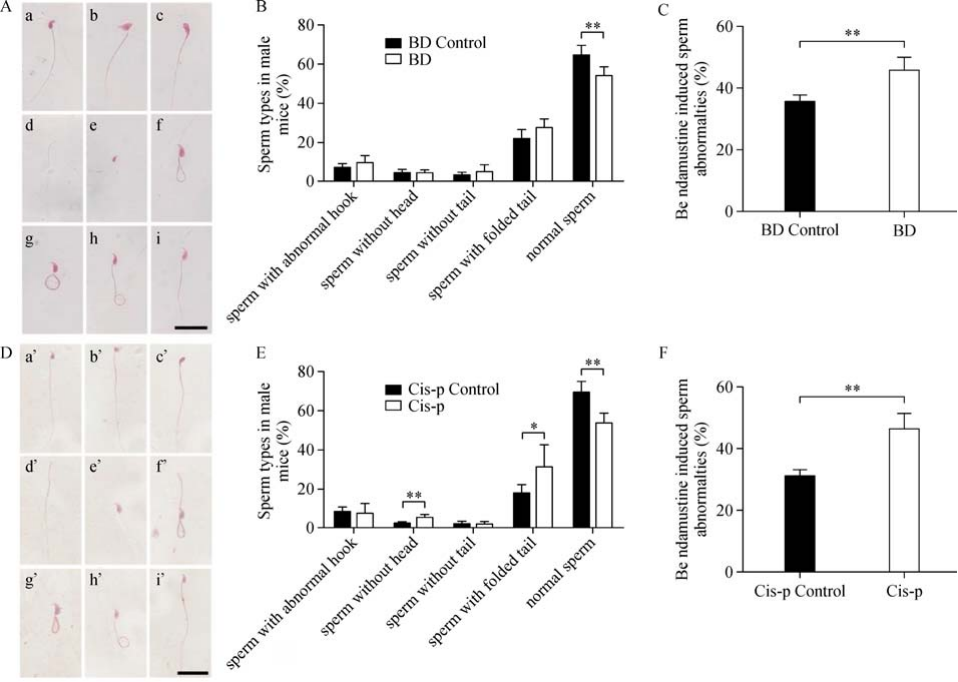
Bendamustine and cisplatin induced sperm morphology abnormalities in mice with hematoxylin-eosin (H&E)staining.

In addition, we observed an increase in the percentage of spermatozoa with morphological abnormalities in bendamustine treated mice, approximately 16% when compared to the bendamustine control mice (***Fig. 7C***). While in the group of cisplatin treated mice, an increase in spermatozoa morphological abnormalities was observed approximately 22% when compared to the cisplatin control mice (***Fig. 7F***). The majority of abnormalities included changes of tail shape as shown in (***Fig. 7A***, ***B***, ***D***, and ***E***).

### Effects of bendamustine on germ cell apoptosis

**Fig.8:**
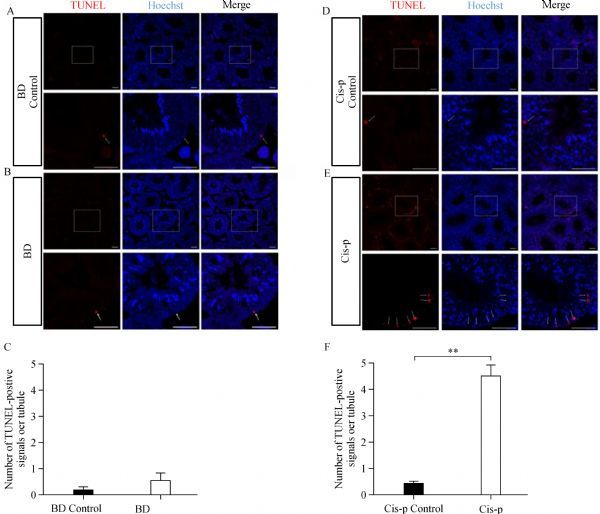
TUNEL assay for apoptosis in the seminiferous tubules.

In the testis cross sections of bendamustine treated mice, TUNEL-positive cells in the seminiferous epithelium were observed, and no marked difference was found when compared to controls (***Fig. 8A***, ***B***, and ***C***). While in the testis cross sections of cisplatin treated mice, the number of TUNEL-positive cells was significantly higher when compared to the cisplatin control mice (***Fig. 8D***, ***E***, and ***F***).

### Effects of bendamustine on serum hormone

**Fig.9:**
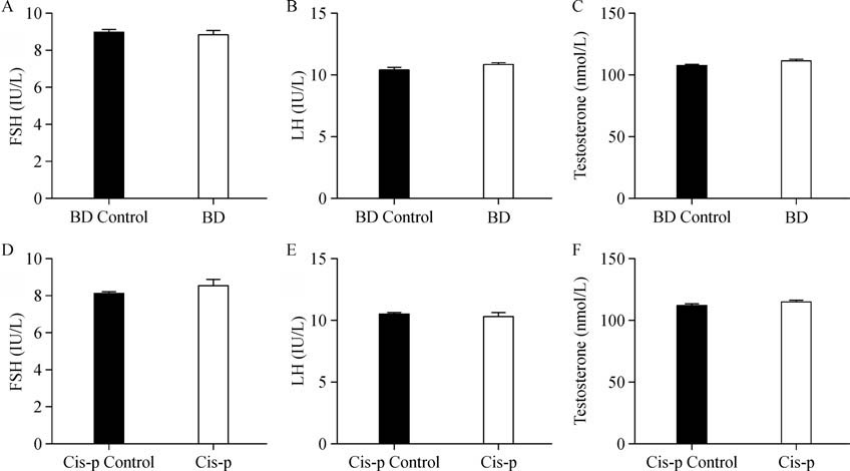
Serum hormone levels of male mice after three weeks of treatment.

We further analyzed serum testosterone, FSH and LH levels in each group of mice after 3 weeks of treatment. Serum testosterone, FSH and LH levels were not significantly altered in bendamustine treated mice and cisplatin treated mice, respectively, when compared to saline treated mice (***Fig. 9***).

## Discussion

Since many of these patients are treated with bendamustine based chemotherapy before and during their reproductive years^[2]^, and cure rates for several types of NHL are high^[6]^, effects on the male reproductive system caused by treatment is a very significant concern. To gain insights on reproductive toxicity of bendamustine, we have assessed sperm morphology, motility, testicular histopathology and other components of spermatogenesis of male mice after treatment. To date, no such comprehensive study has been conducted before.

Generally, the rat is the specie of choice for reproductive toxicity testing^[16]^; however, under some circumstances, data from other mammalian species may be appropriate for incorporation into human health risk assessments; for example, mice in which specific genes have been knocked out may make excellent models for elucidating the mechanisms of toxicant action. Thus, use of other species is likely to become increasingly important as the ability to use mechanistic and molecular genetic information increases^[17]^. In this study, we used mouse model to evaluate the long-term consequences of a three week treatment with bendamustine, mimicking the human clinical regimen for NHL, on male spermatogenesis.

As a marker of the general health of the animals, we recorded the body weights of each male mouse before and after treatment. Our results showed that bendamustine treated mice gained less body weight when compared to vehicle control. Weight loss is a common adverse event of bendamustine, which has been reported in rats and humans^[7^,^18]^. On the other hand, in the experiment, cisplatin treated mice experienced a sharp decrease in body weight, approximately 43%, when compared to vehicle control. In our previous research (pre-test experiment), almost all of the mice which were treated with cisplatin in a higher dose (the dose regimen was chosen based on the standard dose given to humans) died after one week of treatment. Thus, to increase the survival rate of mice during cisplatin treatment we have adjusted the dose in this paper, and to date, several papers which evaluated the reproductive toxicity of cisplatin also reported the low viability of rodents during the treatment. In rodents, previous studies have also reported the impact of cisplatin in the body weight of males after completion of chemotherapy^[12^–^13]^.

Normal testis weight varies only modestly within a given test species^[19]^. This relatively low inter-animal variability suggests that absolute testis weight should be a precise indicator of gonadal injury^[20]^. The testis of male mice was evaluated at the end of the treatment, as previously described. When compared to the bendamustine control mice, significant reduction of testis size (weight) was not found in the bendamustine treated mice. In a sub-chronic study by Horn et al. (1985), significant atrophy of the testes was also not found in male rats that were given bendamustine for 28 days at oral dose levels of 5, 10, 20 or 40 mg/kg/day^[18]^.On the other hand, reduction of the testis size (weight) along with disrupted spermatogenesis are well-known side effects of cisplatin-based chemotherapy^[21]^. Our results showed that cisplatin treatment of male mice significantly decreased the weights of the testes, approximately 36% when compared to the vehicle control. In a sub-chronic by Sawhney et al. (2005), mice that were given 2.5 mg/kg cisplatin for a single round of exposure (one week) demonstrated a 31% decrease in average testis weight^[12]^, while in the rat, a much more severe impact on testis was reported by Bieber et al. (2006), rats that were given 3 mg/kg cisplatin along with bleomycin and etoposide for a total of nine weeks experienced a decrease in testis weight of approximately 50% when compared to controls^[13]^. The decrease in testis weight might indicate loss of cells in seminiferous tubules.

To further investigate impact of these anti-cancer agents on testis of male mice, we evaluated the stages of mouse spermatogenesis using PAS-H staining. Being consistent with the testis size (weight), no clear signs of germ cell degeneration and apparent abnormalities in germ cells and germ cell development were observed in the cross sections of the testis collected from bendamustine treated mice after three weeks of treatment. On the other hand, cisplatin treatment resulted in the appearance of tubules that lacked standard stage-specific cellular associations because of the absence of specific germ cell subpopulations, such as an entire generation of spermatocytes. This could be the consequence of either the loss of those specific populations via apoptosis or a failure of the previous generation to differentiate.

Since the restoration of spermatogenesis depends on the availability of SSCs^[22]^, spermatogenesis is a complex process where many spermatozoa are produced from a small number of SSCs, which occurs during the entire reproductive life of males^[23]^. Similar to other tissue-specific stem cells, SSCs are rare, representing approximately 0.03% of the total germ cells in mice^[24]^. In adult mice, SSCs are restricted to the undifferentiated type A spermatogonia^[25]^. This subset includes A single (As) spermatogonia and their progeny A paired (Apr) and A aligned (Aal) spermat-ogonia^[25^–^26]^. Using whole-mount immunofluorescence staining of PLZF, we compared the number of SSCs both in drug treated mice and in mice of vehicle control. PLZF is the transcription factor of promyelocytic leukemia zinc-finger, which acts by repressing the transcription of genes involved in the differentiation of SSCs^[27^–^28]^. Be consistent with the evaluation of testis histopathology, no difference was found when compared to the number of SSCs at the base of the seminiferous epithelium in bendamustine treated mice and in mice of vehicle control, while cisplatin treated mice underwent a sharp decrease in the number of SSCs when compared to the vehicle control. Published studies have reported the effects of cisplatin based treatment on SSCs of rodents^[29^–^30]^. In rats, Marcon and Zhang have reported that cisplatin based treatment drastically reduced the number of SSCs^[30]^. While in mice, Harman and Richburg found that cisplatin treatment induced an increase in the undifferentiated spermatogonial population and mitotic activity in the recovery period of mice exposed to one cycle treatment^[29]^.

To characterize the nature of germ cell loss after treatment, TUNEL staining was employed after three weeks of treatment. In agreement with the results of testis histopathology, we observed that the incidence of apoptotic cells per tubule was higher in cisplatin treated mice but not in bendamustine treated mice, which may indicate that dramatic depletion of germ cells observed in testis sections of cisplatin treated mice resulted from massive apoptosis.

To further investigate the impact of these alkylating agents on spermatogenesis, we evaluated sperm parameters of each male mouse, including motility, concentration and sperm morphology. Surprisingly, no remarkable differences were found between control and bendamustine treated groups in the parameters of sperm motility and concentration. Bendamustine treatment induced a 10.3% increase of sperm morphology abnormalities when compared to the vehicle control. Analysis of sperm morphology revealed that sperm with folded tails were the most frequent abnormality detected in bendamustine treated mice. In boars, spermatozoa with folded tails have been linked to a droplet migration problem^[31]^. However, the mechanism of bendamustine induced sperm morphology remains to be elucidated. On the other hand, be consistent with previous reports^[13^, ^32]^, cisplatin treatment induced a significant decrease in sperm concentration and motility parameters. Furthermore, an increase of sperm morphology abnormalities has also been detected in cisplatin treated mice when compared to the controls.

Maintenance of spermatogenesis and male fertility is dependent upon the direct and indirect actions of numerous hormones. A large body of literature provides compelling evidence that luteinizing hormone (LH) and follicle stimulating hormone (FSH) are the main regulators of the spermatogenic process in mammals^[33]^. It is known that FSH acts on the Sertoli cell to activate gene expression and signaling pathways that support the process of sperm production and LH acts on the Leydig cell to promote the production of testosterone that is essential for maintaining spermatogenesis^[34]^. To further investigate the effects of these anticancer agents of serum hormones, we compared the serum levels of sex hormones, including FSH, LH, testosterone both in drug treated mice and vehicle control; however, in our experiment, the differences in serum hormones between drug treated mice (including bendamustine treated mice and cisplatin treated mice) and vehicle treated mice were not statistically significant. These results might indicate that bendamustine and cisplatin impact spermatogenesis by targeting specific cell types, for example, germ cells instead of the expression of serum hormones.

In conclusion, the results of the present study indicate that exposure to bendamustine has a deleterious impact on spermatozoa in male mice, resulting in an increase in morphologically abnormal spermatozoa. These data indicate that bendamustine may affect the quality of spermatozoa in patients who have been treated for NHL. In the future, we will investigate the effects of longterm exposure to bendamustine on male fertility and progeny outcome in male mice.

## References

[1] Hochberg J, El-Mallawany NK, Abla O. Adolescent and young adult Non-Hodgkin lymphoma[J]. Br J Haematol, 2016, 173(4): 637–650 . 27071675 10.1111/bjh.14074

[2] Bleyer A, Viny A, Barr R. Cancer in 15- to 29-year-olds by primary site[J]. Oncologist, 2006, 11(6): 590–601 . 16794238 10.1634/theoncologist.11-6-590

[3] Jaffe ES, Harris NL, Stein H, Classification of lymphoid neoplasms: the microscope as a tool for disease discovery[J]. Blood, 2008, 112(12): 4384–4399 . 19029456 10.1182/blood-2008-07-077982 PMC2954680

[4] Patte C, Auperin A, Sebban C, The 15–20 year old patients with NHL treated in France: Data of childhood and adult databases[J]. Ann Oncol, 2005, 16: 61–61.

[5] Hochberg J, Waxman IM, Kelly KM, Adolescent Non-Hodgkin lymphoma and Hodgkin lymphoma: state of the science[J]. Br J Haematol, 2009, 144(1): 24–40 . 19087093 10.1111/j.1365-2141.2008.07393.x

[6] Becker M, Tschechne B, Reeb M, Bendamustine as first-line treatment in patients with advanced indolent Non-Hodgkin lymphoma and mantle cell lymphoma in German routine clinical practice[J]. Ann Hematol, 2015, 94(9): 1553–1558 . 26122866 10.1007/s00277-015-2404-1 PMC4525187

[7] Vacirca JL, Acs PI, Tabbara IA, Bendamustine combined with rituximab for patients with relapsed or refractory diffuse large B cell lymphoma[J]. Ann Hematol, 2014, 93(3): 403–409 . 23955074 10.1007/s00277-013-1879-x PMC3918114

[8] Tageja N, Nagi J. Bendamustine: something old, something new[J]. Cancer Chemother Pharmacol, 2010, 66(3): 413–423 . 20376452 10.1007/s00280-010-1317-x

[9] Tageja N. Bendamustine: safety and efficacy in the management of indolent Non-Hodgkins lymphoma[J]. Clin Med Insights Oncol, 2011, 5(5): 145–156 . 21695099 10.4137/CMO.S6085 PMC3117628

[10] Glantz JC. Reproductive toxicology of alkylating agents[J]. Obstet Gynecol Surv, 1994, 49(10): 709–715 . 7816395 10.1097/00006254-199410000-00026

[11] Hales BF, Barton TS, Robaire B. Impact of paternal exposure to chemotherapy on offspring in the rat[J]. J Natl Cancer Inst Monogr, 2005, 34(34): 28–31 . 15784818 10.1093/jncimonographs/lgi028

[12] Sawhney P, Giammona CJ, Meistrich ML, Cisplatin-induced long-term failure of spermatogenesis in adult C57/Bl/6J mice[J]. J Androl, 2005, 26(1): 136–145 . 15611578

[13] Bieber AM, Marcon L, Hales BF, Effects of chemotherapeutic agents for testicular cancer on the male rat reproductive system, spermatozoa, and fertility[J]. J Androl, 2006, 27(2): 189–200 . 16278370 10.2164/jandrol.05103

[14] Ohmachi K, Niitsu N, Uchida T, Multicenter phase II study of bendamustine plus rituximab in patients with relapsed or refractory diffuse large B-cell lymphoma[J]. J Clin Oncol, 2013, 31(17): 2103–2109 . 23650408 10.1200/JCO.2012.46.5203

[15] Nair AB, Jacob S. A simple practice guide for dose conversion between animals and human[J]. J Basic Clin Pharm, 2016, 7(2): 27–31 . 27057123 10.4103/0976-0105.177703 PMC4804402

[16] Us Epa O O O. Guidelines for reproductive toxicity risk assessment[J]. 1996.

[17] Hayes AW. Principles and Methods of Toxicology, Fifth Edition[J]. Crc Press, 2007.

[18] Horn U, Härtl A, GÜttner J, Toxicity of the alkylating agent bendamustine[J]. Arch Toxicol Suppl, 1985, 8: 504–506 . 3868382 10.1007/978-3-642-69928-3_120

[19] Blazak WF, Ernst TL, Stewart BE. Potential indicators of reproductive toxicity: testicular sperm production and epididymal sperm number, transit time, and motility in Fischer 344 rats[J]. Fundam Appl Toxicol, 1985, 5(6 Pt 1): 1097–1103 . 4092871 10.1016/0272-0590(85)90145-9

[20] Berndtson WE. Methods for quantifying mammalian spermatogenesis: a review[J]. J Anim Sci, 1977, 44(5): 818–833 . 324963 10.2527/jas1977.445818x

[21] Lampe H, Horwich A, Norman A, Fertility after chemotherapy for testicular germ cell cancers[J]. J Clin Oncol, 1997, 15(1): 239–245 . 8996148 10.1200/JCO.1997.15.1.239

[22] Zohni K, Zhang X, Tan SL, The efficiency of male fertility restoration is dependent on the recovery kinetics of spermatogonial stem cells after cytotoxic treatment with busulfan in mice[J]. Hum Reprod, 2012, 27(1): 44–53 . 22082982 10.1093/humrep/der357

[23] Brinster RL, Zimmermann JW. Spermatogenesis following male germ-cell transplantation[J]. Proc Natl Acad Sci U S A, 1994, 91(24): 11298–11302 . 7972053 10.1073/pnas.91.24.11298 PMC45218

[24] Tegelenbosch RA, de Rooij DG. A quantitative study of spermatogonial multiplication and stem cell renewal in the C3H/101 F1 hybrid mouse[J]. Mutat Res, 1993, 290(2): 193–200 . 7694110 10.1016/0027-5107(93)90159-d

[25] de Rooij DG, Russell LD. All you wanted to know about spermatogonia but were afraid to ask[J]. J Androl, 2000, 21(6): 776–798 . 11105904

[26] de Rooij DG. Proliferation and differentiation of spermatogonial stem cells[J]. Reproduction, 2001, 121(3): 347–354 . 11226060 10.1530/rep.0.1210347

[27] Costoya JA, Hobbs RM, Barna M, Essential role of Plzf in maintenance of spermatogonial stem cells[J]. Nat Genet, 2004, 36(6): 653–659 . 15156143 10.1038/ng1367

[28] Buaas FW, Kirsh AL, Sharma M, Plzf is required in adult male germ cells for stem cell self-renewal[J]. Nat Genet, 2004, 36(6): 647–652 . 15156142 10.1038/ng1366

[29] Harman JG, Richburg JH. Cisplatin-induced alterations in the functional spermatogonial stem cell pool and niche in C57/BL/6J mice following a clinically relevant multi-cycle exposure[J]. Toxicol Lett, 2014, 227(2): 99–112 . 24704392 10.1016/j.toxlet.2014.03.019 PMC4041011

[30] Marcon L, Zhang X, Hales BF, Effects of chemotherapeutic agents for testicular cancer on rat spermatogonial stem/progenitor cells[J]. J Androl, 2011, 32(4): 432–443 . 21088230 10.2164/jandrol.110.011601

[31] Bonet S, Briz M, Fradera A, Origin, development and ultrastructure of boar spermatozoa with folded tails and with two tails[J]. Hum Reprod, 1992, 7(4): 523–528 . 1522197 10.1093/oxfordjournals.humrep.a137683

[32] Oshio S, Tomomasa H, Amemiya H, Damaging effects of cisplatin on mouse spermatozoa[J]. Arch Androl, 1990, 24(2): 113–120 . 2327821 10.3109/01485019008986870

[33] Weinbauer GF, Nieschlag E. 4 – Hormonal Control of Spermatogenesis[J]. Molecular Biology of the Male Reproductive System, 1993, 99–142.

[34] Smith LB, Walker WH. Chapter 16–Hormone Signaling in the Testis[J]. Knobil & Neills Physiology of Reproduction, 2015, 637–690.

